# Foot Pain and Morphofunctional Foot Disorders in Patients with Rheumatoid Arthritis: A Multicenter Cross-Sectional Study

**DOI:** 10.3390/ijerph18095042

**Published:** 2021-05-10

**Authors:** María Reina-Bueno, Pedro V. Munuera-Martínez, Sergio Pérez-García, María del Carmen Vázquez-Bautista, Gabriel Domínguez-Maldonado, Inmaculada C. Palomo-Toucedo

**Affiliations:** 1 Department of Podiatry, University of Seville, 41004 Seville, Spain; pmunuera@us.es (P.V.M.-M.); carmenvaz@us.es (M.d.C.V.-B.); gdominguez@us.es (G.D.-M.); ipalomo@us.es (I.C.P.-T.); 2 Department of Health Sciences, University of A Coruña, 15001 A Coruña, Spain; sergio.perez.garcia@udc.es

**Keywords:** rheumatoid arthritis, foot deformities, flatfoot, disability evaluation

## Abstract

Foot problems are highly prevalent in people with rheumatoid arthritis. This study aims to explore the foot morphology, pain and function in rheumatoid arthritis patients and the relation with the time of disease debut. A cross-sectional study was designed. Footprint, the Foot Posture Index, the hallux valgus prevalence, foot pain and function in 66 rheumatoid arthritis patients and the association with time since diagnosis, were recorded. The Foot Function Index, the Manchester Foot Pain and Disability Index, the Visual Analogic Scale, and the Manchester Scale for hallux valgus were administered and analyzed in two groups, with less and more than 10 years of diagnosis of the disease. A high prevalence of pronated (right 36.8% and left 38.6%) and highly pronated (right 15.8% and left 15.8%) feet was observed, as well as an elevated percentage of low arched footprints (right 68.4 and left 66.7%) and hallux valgus (right 59.6% and left 54.4%). Hallux valgus prevalence, toe deformities and Foot Function Index (Functional limitation) factors were significantly associated with the time since RA diagnosed adjusted for the other factors. The adjusted odds ratio of Hallux valgus prevalence was 4.9 (1.2–19.7). In addition, the foot function was diminished, and foot pain was present in most participants. In conclusion, rheumatoid arthritis patients’ feet showed altered morphology and function, and with longer rheumatoid arthritis history, metatarsophalangical stability and foot function, but not pain and global foot posture, were likely to deteriorate.

## 1. Introduction

Rheumatoid arthritis (RA) is a chronic, progressive and inflammatory musculoskeletal disease characterized by symmetric polyarthritis [1]. The prevalence is between 0.3 and 1.5% people worldwide [2,3]. Women are more frequently affected than men, with the highest incidence between the age of 30 and 50 [1].

The essential sign of this autoimmune disease is the destructive capacity of synovium inflammation that causes bone and cartilage erosions and articular deformities in later stages [4]. Inflammation of affected joints causes pain, misalignment, subluxation, decreased range of motion, stiffness and increased mechanical stress [2].

Epidemiology studies suggest that up to 90% of RA patients suffer from foot pain [5]. It is usually present as a persistent inflammatory synovitis affecting and destroying peripheral joints symmetrically [4]. Foot involvement is one of the most worrying problems in patients with RA [3]. The progression of symptoms is related to the duration and severity of the disease [2]. Foot joints are affected in 16% of patients in an initial stage [1]. In 2020, Mochizuki et al. found a prevalence of calloses of 31.2% in RA patients [6]. In a study published in 2017, 91.2% of people diagnosed with RA reported foot problems, 73.8% of them being an articular origin [7].

**Figure 1:**
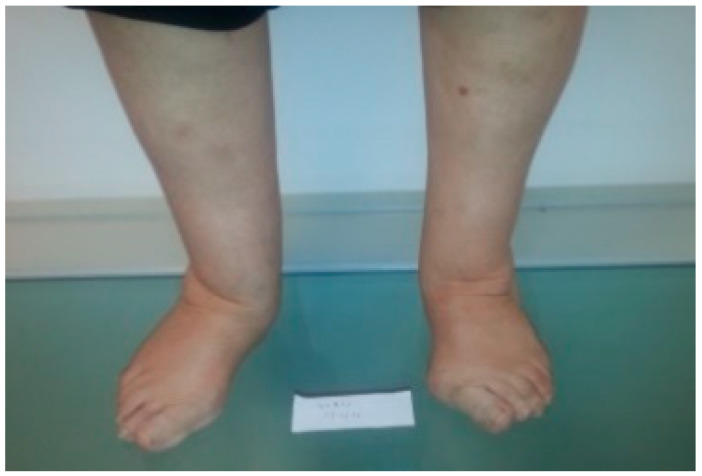
Rheumatoid arthritis feet deformities inspected in the standing position: Hallux abducttus valgus, flatfeet and fibular deviation of metatarsophalangeal joints are observed. Foot posture index evaluation in the anterior view.

The forefoot is commonly involved, especially metatarsophalangeal joints [2] and hypermobility of the first ray [8]. Hallux abductus valgus, lesser toes deformities, metatarsophalangeal joints subluxations, or distal displacement of the plantar pad, are typical disorders [3,4,9,10]. One of the most prevalent pathologies in patients with RA is a rearfoot valgus misalignment, which may be associated with other foot problems (such as those previously mentioned) and symptoms [11]. In addition, flatness of the longitudinal arch is frequently observed, as well as extra-articular manifestations, such as bursitis, nodules, hyperkeratosis and ulcers [7] (Figure 1).

Regarding the lower limb, the symptoms most frequently reported in association with RA are pain [2,3,4,9,10,12,13,14,15], increased plantar pressure [2,4,9,10,12,15], and decreased functional capacity [1,3,12,13,14,15], which may have a negative effect on quality of life [3,7,10,15,16] and risk of falls [7]. Recently, the benefits of custom-made foot orthoses on foot pain have been reported in patients with RA [17,18].

This study aimed to determine the prevalence of pain, HAV, function, posture and disability related to the feet in a group of people with RA. Secondarily, the second objective is to find the association of time since diagnosis with the other items. Time since diagnosis was classified as categorical data with two levels: ≤10 years and >10 years.

## 2. Materials and Methods

### 2.1. Study Design

A cross-sectional study was designed. Authorization was given by the Podiatric Clinical Area of the University of Seville (ID No. INV22/15) and was approved by the Ethic Committee of the Junta de Andalucía (ID No. 20161012141038).

All the participants gave their written consent to be included in the study.

### 2.2. Participants

The participating patients were gathered from the rheumatology unit of the following hospitals: Virgen de Valme, Virgen del Rocío and Virgen Macarena in Seville (Andalusia, southern Spain), and Hospital Básico de la Defensa in Ferrol (Galicia, northern Spain). Participants were also recruited from the following associations of patients: LIRA (Andalusian Rheumatologic League), AJEREA (Provincial Spondylitis and Arthritis Association) and ASEPAR (Sevillian Association of Patients with Rheumatoid Arthritis). The data collection was carried out at the Clinical Area of Podiatry of the University of Seville and at the Podiatric Clinic of the University of A Coruña from January 2016 to October 2017.

The inclusion criteria were patients aged over 18 years diagnosed with RA according to the criteria of the American Rheumatism Association of 1987 [19], with foot involvement diagnosed by a rheumatologist. Participants were excluded if they presented in an acute symptomatic flare or they needed to use walking assistance. Other exclusion criteria were neurological problems, malignant process, cognitive deterioration, pregnancy, previous foot surgery and the presence of a foot wound.

### 2.3. Measurements

Clinical and demographic data were collected, including age, gender, weight, height, years of development of the disease, foot pain, and disability related to foot pain. AR patients were explored and Foot Posture Index (FPI) [20] and Manchester scale for hallux valgus were recorded [21].

**Figure 2:**
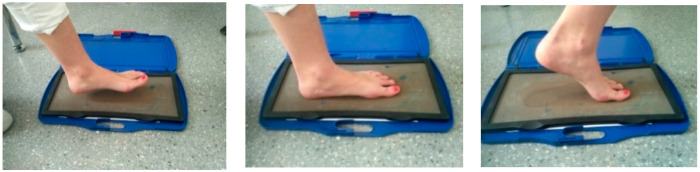
The patient walks on the ink footprint device to take the foot print. The progression of the step is shown during the standing phase. The other limb progresses from back to forward.

Foot Posture Index is a clinical tool to assess foot posture during weight bearing by collecting data from six criteria (palpation of the head of the talus; curvature difference above and below the peroneal malleolus; position of the calcaneus in the frontal plane; prominence in the talo-navicular joint; the medial longitudinal arch’s congruence; and abduction/adduction of the forefoot from the posterior view), which are scored from +2 to −2. Values between −12 and +12 may be obtained in order to classify feet in neutral (0 to +5), supinated (−4 to −1), highly supinated (−12 to −5), pronated (+6 to +9) or highly pronated positions [20]. Ink footprints were obtained from all participants (Figure 2).

**Figure 3:**
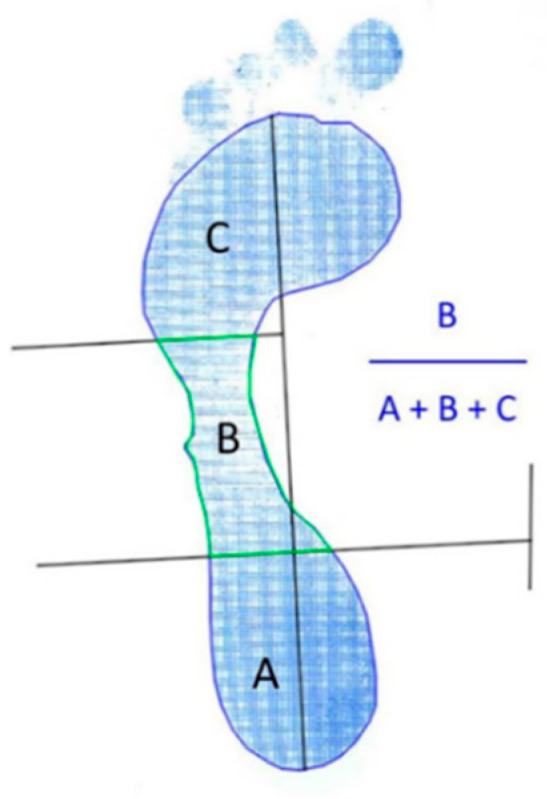
Footprint measurements: Arch Index. (**A**) = rearfoot plantar area, (**B**) = midfoot plantar area, (**C**) = forefoot plantar area excluding the toes.

AutoCad^®^ software (AutoCAD 2019; Autodesk Inc, San Rafael, CA, USA) was used to calculate the Arch Index in the footprint [22]. The Arch Index was obtained through the proportion between the area of the central part of the footprint (**B**) and its total area (**A**) + (**B**) + (**C**). Results less than 0.21 correspond to high arched feet, between 0.21 and 0.26 suggest normal feet, and higher than 0.26 correspond to low arched feet [23] (Figure 3).

Pain was measured using the visual analogue scale (VAS) ranging from 0 (no pain) to 10 cm (unbearable pain). The pain days were also recorded, as the number of days in which the patient felt foot pain in the previous week, assigning a whole number between 0 and 7 [24].

Foot functionality was measured through the Foot Function Index (FFI), a questionnaire of 23 items divided into three domains (foot pain, disability and functional limitation), with values ranging between 0 and 100, where the higher values correspond to greater pain, disability and limitation [25].

Disability related to foot pain was measured using the Manchester Foot Pain and Disability Index (MFPDI). The values of this index range from 0 to 38, with higher values corresponding to greater disability [26].

### 2.4. Data Analysis

The analysis of the data was carried out using the statistical software IBM SPSS Statistics 24 ^®^ (IBM, Armonk, NY, USA). The descriptive data provided the mean values and the standard deviations for quantitative variables, and the absolute (*n*) and relative (%) frequencies for categorical variables.

Inferential analysis was carried out taking into account a confidence level of 95% (*p* values lower than 0.05 were considered statistically significant, but *p* values higher than 0.05 and lower than 0.10 were interpreted as showing a trend towards significance).

The Shapiro–Wilk test was applied to the data from the quantitative variables to decide whether a parametric or non-parametric test should be used. Comparisons between patients who had RA for 10 years or less and patients who had RA for more than 10 years were made using the Student T test for independent samples.

A comparison was made using the chi-square test to determine the degree of dependence between the variables. A multivariate binary logistic regression model was then carried out, only including the variables whose *p*-value was lower than 0.2. The method used was the method of backward elimination (conditional). For the variables that were statistically significant, the odds ratio (OR) was calculated.

## 3. Results

Sixty-six patients (11 men and 44 women) with a RA diagnosis were included in this study, with a mean age of 60.19 ± 1.51 years, BMI 26.93 ± 0.69 kg/m^2^, and mean time from diagnosis 14.42 ± 1.52 years.

Mean (± SD) values of FPI were 5.21 ± 0.56 for the right foot and 5.28 ± 0.54 for the left foot.

Other characteristics of the participants’ feet are summarized in Table 1.

Regarding perceived foot pain, participants reported a mean (± SD) value of 6.53 ± 1.98 by means of VAS. Data related to pain and disability obtained via FFI and MFPDI questionnaires are shown in Table 2.

Table 3 shows the descriptive values and comparisons of the quantitative variables between those participants with 10 years or less with RA and those with more than 10 years with RA. No significant differences were observed between groups.

Participants’ self-reported general characteristics, participants’ self-reported and observed foot problems, and categorized results of the scales used in both groups are shown in Table 4. Results of the univariate and multivariate binary logistic regression, with odds ratios and *p* values, are included in this table. Note that variables with *p*-values lower than 0.2 in the univariate analysis were included in the multivariate one. The dependent variable was the years with RA of participants (equal to or less than 10 years, and more than 10 years). Toe deformities is the variable that showed more difference between groups, with an OR of 13.2. An unexplainable finding is the fact that OR for FPI right foot was 0.0 in the multivariate analysis, showing worse FPI categorization values in those patients with less years of RA.

## 4. Discussion

The main objective of the present study was to describe the type of foot, the morphology of the footprint and the presence and grade of hallux valgus in a group of people diagnosed with RA. Secondarily, the foot pain, foot function and disability, were assessed. According to the data obtained, there was a high prevalence of pronated and highly pronated feet, flattened arches and hallux valgus. Moreover, decreased foot function and high foot pain values were also observed.

Due to the high prevalence of feet symptoms, reflecting the underlying pathologies that cause pain, deformities and biomechanical alterations, podiatry treatment may be useful. The use of custom-made insoles has proven to be effective on the pain of patients with other rheumatologic diseases. For this reason, it is important that the podiatrist is part of a multidisciplinary team.

According to the FPI scale, more than half of the patients had a pronated or highly pronated foot posture. Only 10% had a supinated or very supinated foot posture. Similar results were reported by Biscontini et al. [27] who studied the foot structure of 78 patients with RA using the FPI and observed that 65.4% of them presented with a pronated posture, and 34.6% had a supinated posture. In the present sample, the frequency of pronated and supinated feet was lower. This may be explained by the fact that, in this study, only patients with painful symptoms were included and not those with exclusively structural alterations in their feet. González-Fernández et al. [28] conducted a study to determine foot problems in people with RA (*n* = 62), compared with a control group (*n* = 74). The prevalence of alterations was higher in the affected participants, which resulted in a FPI pronated position of 47.6% compared to 24.5% in the control group. The FPI supinated position was 23.4% in the RA group versus 10.9% in the control group. This value was similar with regard to the pronated position, but the supinated feet in our sample showed a lower percentage. In any case, studies confirm that one of the most prevalent foot pathologies in RA is rearfoot valgus, corresponding to the pronated position [11]. These values are much higher than those described in a normal population by Redmond et al. [29].

The type of footprint via the Arch Index showed a low longitudinal arch of the foot in more than 66% of cases. Pita et al. [30] established the prevalence of flat feet in adults aged over 40 years, setting it at around 26% in a randomized normal population, using the Clark’s angle and the Arch Index. Some authors sustain that flattening of the longitudinal arch is a problem associated with RA [31] and that this prevalence is higher than in the non-RA population.

A large variability of measurement methods and data has been reported in the literature. Bal et al. [31] conducted a study with radiographs in 78 RA patients and 76 healthy people. They determined a prevalence of flatfoot of 80.1% via measuring the calcaneal pith angle, which was also high in the control group (44.7%). However, using the same radiological measurements, the values obtained by Karatepe et al. were much lower in both groups, establishing that 36.3% of the RA patients had flat feet, a little lower percentage than in the healthy individuals [32]. We cannot explain those differences in results as the samples were similar in terms of age, gender and geographic area, as both were conducted in Turkey.

On the other hand, Rojas-Villarraga et al. [33] determined by visual examination the prevalence of different foot problems related to RA, reporting that in 42% of patients, the longitudinal arch was flattened. Similarly, by visual assessment of the footprint on a podoscope, González-Fernández et al. [28] affirmed that the footprint was abnormal in 66.9%, without specifying the type of footprint, being lower in the control group. Our values show some concordance with the values found by those authors.

In a review published by Stolt et al. in 2017, flatfoot in RA patients had a prevalence ranging from 11% to 42.1% [34]. This is lower than that reported in the participants of the present study, although only one article of those meeting the inclusion criteria used the FPI and none used the Arch Index. Therefore, the different measurement methods may explain the variability of results in similar populations.

RA is related to other foot alterations such as hallux abductus valgus [3,4,9,10]. In the patients that participated in this study, hallux abductus valgus had a prevalence of about 54%. This deformity is often present in healthy adults, especially women, with an estimated prevalence of 23% [35]. Some authors have quoted higher figures (84.6%), employing the Manchester scale for hallux abductus valgus (23.1% mild, 37.2% moderate, 24.4% severe) [27]. Total frequency is similar to that obtained in this study, although the percentage of moderate and severe cases is lower. Using radiographic measurements, some authors established the prevalence of hallux abductus valgus in 64.1% [31] and 62.5% [32]. The latter study reported that it was the most prevalent foot deformity. These results agree with those found in this study.

Although some authors report lower figures (28), we agree that hallux abductus valgus is one of the most common structural problems in patients with RA, affecting between 35–65.3% of patients [34]. According to what has been found in the literature, and considering the data obtained, the values of prevalence are disparate. It would be necessary to accurately determine the prevalence of this deformity in RA. However, co-factors such as age, sex or the use of inappropriate footwear are elements that have been shown to have an influence on the presence or severity of hallux abductus valgus [36], and variability can be found in certain societies, geographical distribution, or ethnic groups.

In addition to morphological alterations, RA patients experiment foot pain and impairment of foot function. Authors who previously measured the FFI in people with RA concluded that foot function was affected [31]. In 2018, De Andrade et al. [37] conducted a cross-sectional study to determine the foot function in a group of 100 RA patients compared to 100 controls. In subjects with RA, the mean values of the different domains of the FFI questionnaire were 19 on activity limitation, 53.3 on disability, 51.4 on pain, and the total punctuation was 42.3 points. These values are like those obtained in the present study. Apart from the FFI, the Foot and Ankle Outcome Score and the most widespread, the Leeds Foot Impact Scale, are used to determine the degree of disability and function of the lower limb, and results from those scales also confirm the findings of functional impairment in affected individuals [38]. Morpeth et al. [39] reported similar results with the Leeds Foot Impact Scale and correlated the biomechanics impairment to risk of falls, foot pain and disability in people with RA. VAS 100 was used to quantify foot pain and, compared to a control group, RA participants showed higher severity of pain and disability, although VAS values were lower than those obtained in this study. Stewart et al. [40] also found higher values of foot pain in 21 patients with RA than in 19 controls. However, these values were about 3.38, which is lower than that observed in the participants of the present study.

According to the results of this study, foot involvement in RA patients was of great relevance. Synovial inflammation can affect all joints of the foot, leading to increased pain, decreased foot function and the development of deformities. Involvement of the first metatarsophalangeal joint is commonly associated with hallux abductus valgus [41]. Inflammation of the subtalar and midtarsal joints leads to pronation of the foot and a flattened arch. However, the DAS 28, the most commonly used index to assess RA activity and make therapeutic decisions, does not include the count of swollen joints in the feet among its parameters [42]. Due to the relevance of these problems, in the authors’ opinion it is necessary to take them into account in the overall assessment and treatment of these patients.

Orthopaedic treatment in RA patients must be custom-made [17]; however, conservative treatments such as splints, insoles or orthoses are not effective in restoring hallux realignment, but are useful in relieving symptomatology and maintaining post-surgical correction [43]. An adequate insole reduces forefoot pain. The use of current orthopedic footwear has opened up new treatment possibilities [44]. Conservative orthopedic measures can prevent deterioration of hallux valgus only at an early stage of the disease. As for surgical techniques, more than 150 different surgical procedures are described in the literature, which can be reduced to a few common procedures. These depend on the manifestation of the bunion, as well as on the associated foot and ankle pathologies [45].

Further research will focus on comparing the podiatric conditions of other rheumatologic pathologies in order to determine whether there is a common podiatric condition in diseases of autoimmune origin. A control group will also be included since it is a weakness of this study.

## 5. Conclusions

To conclude, RA patients who were included in this study showed a high frequency of pronated foot position, which leads to a valgus rearfoot, flattened longitudinal arch and hallux valgus. In this group, the degree of pain was high and foot function was altered. Participants who showed more years since the diagnosis of RA presented with a higher percentage of toe deformities.

## Figures and Tables

**Table 1:** Participants’ feet characteristics.

Foot Characteristics	*n* (%)	Mean ± Standard Deviation
Hallux valgus (right foot)	Mild = 17 (29.8) Moderate = 11 (19.3) Severe = 6 (10.5) TOTAL = 34 (59.6)	
Hallux valgus (left foot)	Mild = 17 (29.8) Moderate = 9 (15.8) Severe = 5 (8.8) TOTAL = 31 (54.4)	
Toe deformities	48 (85.7)	
Arch Index (right foot)	Normal = 12 (21.1) Low arched = 39 (68.4) High arched = 6 (10.5)	0.27 ± 0.00
Arch Index (left foot)	Normal = 12 (21.1) Low arched = 38 (66.7) High arched = 7 (12.3)	0.27 ± 0.00
FPI (right foot)	Normal = 21 (36.8) Pronated = 21 (36.8) Highly pronated = 9 (15.8) Supinated = 3 (5.3) Highly supinated = 3 (5.3)	5.21 ± 0.56
FPI (left foot)	Normal = 21 (36.8) Pronated = 22 (38.6) Highly pronated = 9 (15.8) Supinated = 3 (5.3) Highly supinated = 2 (3.5)	5.28 ± 0.54

Hallux abductus diagnosis according to the Manchester Scale. Arch Index results using footprints according to Cavangh el al. Foot Posture Index score for right and left foot.

**Table 2:** Pain and foot disability results.

Variables	Mean ± Standard Deviation
Visual Analogue Scale	6.17 ± 0.33
Days with foot pain	5.64 ± 0.31
FFI (pain)	65.80 ± 2.52
FFI (disability)	56.07 ± 3.68
FFI (activity limitation)	16.26 ± 2.69
FFI total	51.26 ± 2.57
MFPDI (function)	12.10 ± 0.64
MFPDI (personal appearance)	1.22 ± 0.19
MFPDI (pain)	6.50 ± 0.33
MFPDI (work)	2.38 ± 0.21
MFPDI total	22.19 ± 1.10

Mean values of foot pain measured by VAS. Number of days per week with perceived foot pain. Foot Function Index values according to different dimensions and total score. Each dimension value and total score of Manchester Foot Pain and Disability Index.

**Table 3:** Descriptive values and comparisons of the quantitative variables between those participants with 10 years or less with RA and those with more than 10 years with RA.

Variables	≤10 Years *n* = 27 (47.4%)	>10 Years *n* = 30 (52.6%)	*p*
Visual analogue scale	6.6 ± 2.0	6.6 ± 2.8	0.659
Days with foot pain	5.5 ± 2.3	5.8 ± 2.1	0.619
Toe deformities	20 (41.7%)	28 (58.3%)	0.085 *
Arch index (right foot)	0.25 ± 0.03	0.30 ± 0.05	0.056 *
Arch index (left foot)	0.27 ± 0.05	0.27 ± 0.05	0.173
FPI (right foot)	6.9 ± 5.4	7.6 ± 3.9	0.191
FPI (left foot)	7.1 ± 5.1	7.2 ± 3.9	0.260
FFI (Pain)	65.5 ± 18.2	66.1 ± 20.1	0.898
FFI (Disability)	54.9 ± 26.2	57.1 ± 29.7	0.763
FFI (Functional limitation)	14.3 ± 18.1	23.7 ± 21.4	0.079 *
FFI (Total)	49.6 ± 18.6	52.8 ± 20.3	0.544
MFPDI (function)	11.4 ± 4.9)	12.7 ± 4.8	0.310
MFPDI (personal appearance)	1.1 ± 1.5	1.4 ± 1.5	0.459
MFPDI (pain)	6.7 ± 2.8	6.3 ± 2.3	0.588
MFPDI (work)	2.3 ± 4.7	2.5 ± 1.6	0.583
MFPDI (Total)	21.8 ± 9.0	22.5 ± 7.8	0.748

* These differences show a trend towards significance.

**Table 4:** Participants’ characteristics, foot problems, and values for different scales employed in patients with 10 years or less with RA and those with more than 10 years with RA.

Outcomes	Years with RA					Multivariate	
	≤10 Years		>10 Years		*p*	OR	*p*
	*n* = 27 (47.4%)		*n* = 30 (52.6%)				
	*n*	%	*n*	%			
Toe deformities	21	77.8	28	93.3	0.095 *		
**Manchester Scale for hallux valgus (right foot)**					0.182		
A	14	51.9	8	27.6			
B	7	25.9	10	34.5			
C	5	18.5	6	20.7			
D	1	3.7	5	17.2			
**Manchester Scale for hallux valgus (left foot)**					0.344		
A	15	55.6	11	36.7			
B	8	29.6	9	30			
C	3	11.1	6	20			
D	1	3.7	4	13.3			
**Hallux valgus (yes or not)** #	13	48.1	21	72.4	0.056 *	4.9 (1.2–19.7)	0.024 **
**Arch index (right foot)**					0.140 *		
Low arch	15	55.6	24	80			0.095 ***
High arch	4	14.8	2	6.7			0.29
Normal arch	8	29.6	4	13.3			Ref.
**Arch index (left foot)**					0.816		
Low arch	17	63	21	70			
High arch	4	14.8	3	10			
Normal arch	6	22.2	6	20			
**FPI right foot**					0.088 *		
Normal	7	25.9	14	46.7			
Pronated	15	55.6	15	50			
Supinated	5	18.5	1	3.3			
**FPI left foot**					0.134 *		
Normal	7	25.9	14	46.7			Ref.
Pronated	16	59.3	15	50			0.056 ***
Supinated	4	14.8	1	3.3			0.066 ***
	Mean ± SD; Median (IQR)		Mean ± SD; Median (IQR)				
Visual analogue scale	6.0 ± 2.5; 6 (5–7)		6.3 ± 2.6; 6.5 (4.8–8.3)		0.699		
Days with foot pain	5.5 ± 2.3; 7 (3.8–7)		5.8 ± 2.1; 7 (4.8–7)		0.594		
FFI (Pain)	65.5 ± 18.2; 66.7 (52.2–84.3)		66.1 ± 20.1; 71.3 (54–81.2)		0.565		
FFI (Disability)	54.9 ± 26.2; 55.6 (28.9–80)		57.1 ± 29.7; 66.7 (32.5–80)		0.643		
FFI (Functional limitation)	14.3 ± 18.1; 12 (0–16)		23.7 ± 21.4; 18.8 (9.4–34.5)		0.050 *		
FFI (Total)	49.6 ± 18.6; 52.2 (35.7–66.2)		52.8 ± 20.3; 57.7 (39.1–64.6)		0.31		
MFPDI (function)	11.4 ± 4.9; 12 (8–15)		12.7 ± 4.8; 13.5 (9–17)		0.31		
MFPDI (personal appearance)	1.1 ± 1.5; 0 (0–2)		1.4 ± 1.5; 1 (0–2.3)		0.378		
MFPDI (pain)	6.7 ± 2.8; 7 (6–9)		6.3 ± 2.3; 6 (4–8)		0.472		
MFPDI (work)	2.3 ± 4.7; 2 (1–4)		2.5 ± 1.6; 2.5 (1.5–4)		0.582		
MFPDI (Total)	21.8 ± 9.0; 23 (13–30)		22.5 ± 7.8; 22 (18.5–30)		0.923		

* *p*-value lower than 0.2 according to squared chi test, that were included in the multivariate analysis of binary logistic regression; ** *p*-value lower than 0.05; *** *p*-value lower than 0.1, showing a trend towards significance. FPI: Foot Posture Index; FFI: Foot Function Index, MFPDI: Manchester Foot Pain and Disability Index. # Hallux valgus through the Manchester Scale was reduced to only one variable (to have hallux valgus or not) for the multivariate analysis.

## References

[1] Moreira E., Jones A., Oliveira H., Jennings F., Fernandes A., Natour J. (2016). Effectiveness of insole use in rheumatoid feet: A randomized controlled trial. Scand. J. Rheumatol..

[2] Novak P., Burger H., Tomsic M., Marincek C., Vidmar G. (2009). Influence of foot orthoses on plantar pressures, foot pain and walking ability of rheumatoid arthritis patients—A randomised controlled study. Disabil. Rehabil..

[3] Bagherzadeh Cham M., Ghasemi M.S., Forogh B., Sanjari M.A., Zabihi Yeganeh M., Eshraghi A. (2013). Effect of rocker shoes on pain, disability and activity limitation in patients with rheumatoid arthritis. Prosthet. Orthot. Int..

[4] Cuesta-Calleja R., Polo-García A., González-Fernández M.L. (2018). Evidencia cientifica del tratamiento ortopodologico en pie afectado por artritis reumatoide. Rev. Int. Cienc. Podol..

[5] Otter S.J., Lucas K., Springett K., Moore A., Davies K., Young A., Walker-Bone K. (2011). Comparison of foot pain and foot care among rheumatoid arthritis patients taking and not taking anti-TNFalpha therapy: An epidemiological study. Rheumatol. Int..

[6] Mochizuki T., Yano K., Ikari K., Hiroshima R., Ishibashi M., Okazaki K. (2020). Relationship of callosities of the forefoot with foot deformity, Health Assessment Questionnaire Disability Index, and joint damage score in patients with rheumatoid arthritis. Mod. Rheumatol..

[7] Wilson O., Hewlett S., Woodburn J., Pollock J., Kirwan J. (2017). Prevalence, impact and care of foot problems in people with rheumatoid arthritis: Results from a United Kingdom based cross-sectional survey. J. Foot Ankle Res..

[8] Biz C., Favero L., Stecco C., Aldegheri R. (2012). Hypermobility of the first ray in ballet dancer. Muscles Ligaments Tendons J..

[9] Riskowski J., Dufour A.B., Hannan M.T. (2011). Arthritis, foot pain and shoe wear: Current musculoskeletal research on feet. Curr. Opin. Rheumatol..

[10] Santos D., Cameron-Fiddes V. (2014). Effects of Off-the-Shelf Foot Orthoses on Plantar Foot Pressures in Patients with Early Rheumatoid Arthritis. J. Am. Podiatr. Med. Assoc..

[11] Barn R., Brandon M., Rafferty D., Sturrock R.D., Steultjens M., Turner D.E., Woodburn J. (2014). Kinematic, kinetic and electromyographic response to customized foot orthoses in patients with tibialis posterior tenosynovitis, pes plano valgus and rheumatoid arthritis. Rheumatology.

[12] Gatt A., Formosa C., Otter S. (2016). Foot orthoses in the management of chronic subtalar and talo crural joint pain in rheumatoid arthritis. Foot.

[13] Tenten-Diepenmaat M., Leeden Van Der M., Vliet Vlieland T., Roorda L.D., Dekker J., Gijon-Nogueron G., Ramos-Petersen L., Garcia-Mayor S., Morales-Asencio J.M. (2017). HPR the effectiveness of therapeutic footwear in patients with rheumatoid arthritis: A systematic review and meta-analysis. Ann. Rheum. Dis..

[14] Rome K., Clark H., Gray J., McMeekin P., Plant M., Dixon J. (2017). Clinical effectiveness and cost-effectiveness of foot orthoses for people with established rheumatoid arthritis: An exploratory clinical trial. Scand. J. Rheumatol..

[15] Tenten-Diepenmaat M., Dekker J., Steenbergen M., Huybrechts E., Roorda L.D., van Schaardenburg D., Bus S.A., van der Leeden M. (2016). In-shoe plantar pressure measurements for the evaluation and adaptation of foot orthoses in patients with rheumatoid arthritis: A proof of concept study. Gait Posture.

[16] Santos D. (2015). The Effects of off-the-Shelf Foot Orthoses on the Quality of Life of Patients Diagnosed with Early Rheumatoid Arthritis. Clin. Res. Foot Ankle.

[17] Reina-Bueno M., Vázquez-Bautista M.d.C., Pérez-García S., Rosende-Bautista C., Sáez-Díaz A., Munuera-Martínez P.V. (2019). Effectiveness of custom-made foot orthoses in patients with rheumatoid arthritis: A randomized controlled trial. Clin. Rehabil..

[18] Reina-Bueno M., Ballesteros-Mora M., Rodríguez-Moreno I., Vázquez-Bautista C., Pérez-García S., Rosende-Bautista C., Munuera-Martínez P.V. (2018). Efecto de las ortesis plantares hechas a medidas versus placebo en pacientes con artritis reumatoide: Ensayo clínico aleatorizado. Estudio piloto. Rev. Española Podol..

[19] Aletaha D., Neogi T., Silman A.J., Funovits J., Felson D.T., Bingham C.O., Birnbaum N.S., Burmester G.R., Bykerk V.P., Cohen M.D. (2010). 2010 rheumatoid arthritis classification criteria: An American College of Rheumatology/European League Against Rheumatism collaborative initiative. Ann. Rheum. Dis..

[20] Redmond A.C., Crosbie J., Ouvrier R.A. (2006). Development and validation of a novel rating system for scoring standing foot posture: The Foot Posture Index. Clin. Biomech..

[21] Garrow A.P., Papageorgiou A., Silman A.J., Thomas E., Jayson M.I., Macfarlane G.J. (2001). The grading of hallux valgus. The Manchester Scale. J. Am. Podiatr. Med. Assoc..

[22] Reina M., Lafuente G., Munuera P.V. (2013). Effect of custom-made foot orthoses in female hallux valgus after one-year follow up. Prosthet Orthot Int..

[23] Cavanagh P.R., Rodgers M. (1987). Technical Note the Arch Index: A Useful Measure. J. Biomech. Biomech..

[24] Landorf K.B., Radford J.A. (2008). Minimal important difference: Values for the Foot Health Status Questionnaire, Foot Function Index and Visual Analogue Scale. Foot.

[25] Paez-Moguer J., Budiman-Mak E., Cuesta-Vargas A.I. (2014). Cross-cultural adaptation and validation of the Foot Function Index to Spanish. Foot Ankle Surg..

[26] Gijon-Nogueron G., Ndosi M., Luque-Suarez A., Alcacer-Pitarch B., Munuera P.V., Garrow A., Redmond A.C. (2014). Cross-cultural adaptation and validation of the Manchester Foot Pain and Disability Index into Spanish. Qual. Life Res..

[27] Biscontini D., Bartoloni Bocci E., Gerli R. (2009). Analysis of Foot Structural Damage in Rheumatoid Arthritis: Clinical Evaluation by Validated Measures and Serological Correlations. Reumatismo.

[28] González-Fernández M.L., Valor L., Morales-Lozano R., Hernández-Flórez D., López-Longo F.J., Martínez D., González C.M., Monteagudo I., Martínez-Barrio J., Garrido J. (2016). To what extent is foot pain related to biomechanical changes and ultrasound-detected abnormalities in rheumatoid arthritis?. Clin. Exp. Rheumatol..

[29] Redmond A.C., Crane Y.Z., Menz H.B. (2008). Normative values for the Foot Posture Index. J. Foot Ankle Res..

[30] Pita-Fernandez S., Gonzalez-Martin C., Aalonso-Tajes F., Seoane-Pillado T., Pertega-Diaz S., Perez-Garcia S., Seijo-Bestilleiro R., Balboa-Barreiro V. (2017). Flat foot in a random population and its impact on quality of life and functionality. J. Clin. Diagn. Res..

[31] Bal A., Aydog E., Aydog S.T., Cakci A. (2006). Foot deformities in rheumatoid arthritis and relevance of foot function index. Clin. Rheumatol..

[32] Göksel Karatepe A., GüNaydin R., Adibelli Z.H., Kaya T., DuruöZ E. (2010). Foot deformities in patients with rheumatoid arthritis: The relationship with foot functions. Int. J. Rheum. Dis..

[33] Rojas-Villarraga A., Bayona J., Zuluaga N., Mejia S., Hincapie M.E., Anaya J.M. (2009). The impact of rheumatoid foot on disability in Colombian patients with rheumatoid arthritis. BMC Musculoskelet. Disord..

[34] Stolt M., Suhonen R., Leino-Kilpi H. (2017). Foot health in patients with rheumatoid arthritis—A scoping review. Rheumatol. Int..

[35] Nix S., Smith M., Vicenzino B. (2010). Prevalence of hallux valgus in the general population: A systematic review and meta-analysis. J. Foot Ankle Res..

[36] Valero-Salas J., Palomo-Toucedo I.C., Munuera-Martínez P.V., Munuera-Martínez P.V. (2012). El Hallux Abductus Valgus. El primer Radio: Biomecánica y Ortopodología.

[37] De Andrade A.P., Inoue E.N., Nisihara R., Skare T.L. (2018). Foot function in rheumatoid arthritis patients: A cross-sectional study. Clin. Rheumatol..

[38] Carter K., Lahiri M., Cheung P.P., Santosa A., Rome K. (2016). Prevalence of foot problems in people with inflammatory arthritis in Singapore. J. Foot Ankle Res..

[39] Morpeth T., Brenton-Rule A., Carroll M., Frecklington M., Rome K. (2016). Fear of falling and foot pain, impairment and disability in rheumatoid arthritis: A case-control study. Clin. Rheumatol..

[40] Stewart S., Carroll M., Brenton-Rule A., Keys M., Bell L., Dalbeth N., Rome K. (2018). Region-specific foot pain and plantar pressure in people with rheumatoid arthritis: A cross-sectional study. Clin. Biomech..

[41] Singh D., Biz C., Corradin M., Favero L. (2016). Comparison of dorsal and dorsomedial displacement in evaluation of first ray hypermobility in feet with and without hallux valgus. Foot Ankle Surg..

[42] Van Riel P.L.C.M., Renskers L. (2016). The Disease Activity Score (DAS) and the Disease Activity Score using 28 joint counts (DAS28) in the management of rheumatoid arthritis. Clin. Exp. Rheumatol..

[43] Fuhrmann R.A., Rippel W., Traub A. (2017). Konservative Therapie des Hallux-valgus-Syndroms: Was kann man mit Schienen und Einlagen erreichen?. Orthopade.

[44] Stukenborg-Colsman C. (2017). Hallux valgus: Konservative und operative Therapie. Orthopade.

[45] Zirngibl B., Grifka J., Baier C., Götz J. (2017). Hallux valgus: Ätiologie, diagnostische und therapeutische Prinzipien. Orthopade.

